# Recent Preoperative Lumbar Epidural Steroid Injection Is an Independent Risk Factor for Incidental Durotomy During Lumbar Discectomy

**DOI:** 10.1177/2192568219833656

**Published:** 2019-03-21

**Authors:** Lawal A. Labaran, Varun Puvanesarajah, Sandesh S. Rao, Dennis Chen, Francis H. Shen, Amit Jain, Hamid Hassanzadeh

**Affiliations:** 1 University of Virginia, Charlottesville, VA, USA; 2 Johns Hopkins Hospital, Baltimore, MD, USA

**Keywords:** spine surgery, lumbar discectomy, epidural, corticosteroid, durotomy

## Abstract

**Study Design:**

Retrospective review.

**Objective:**

To investigate the association between lumbar epidural steroid injection (LESI) and incidental durotomy (ID) in patients with a diagnosis of disc herniation undergoing a primary discectomy.

**Methods:**

A Medicare patient database was queried for patients between the ages of 65 and 85 years who underwent a primary lumbar discectomy for a diagnosis of lumbar disc herniation or degeneration from 2008 to 2014. Our main cohort of 64 849 patients was then divided into 2 groups: patients who experienced a dural tear (N = 2369) and our matched (age, gender, and history of diabetes) control cohort of patients who did not (N = 62 480). All patients who had a history of LESI were further identified and stratified into 4 subgroups by duration between LESI and discectomy (<3 months, 3-6 months, 6 months to 1 year, and overall), and a comparison of the relative incidence of ID was made among these subgroups. A multivariate logistic regression analysis was employed to determine the relationship between LESI and ID.

**Results:**

Overall incidence of ID was 3.7%. There was a significant difference in incidence of LESI (27.1% vs 35.0%, *P* < .001) between our control and ID groups. An adjusted odds ratio (OR) showed that prior LESI within 3 to 6 months (OR 1.47, 95% CI 1.20-1.81, *P* < .001) and within less than 3 months (OR 1.46, 95% CI 1.24-1.72, *P* < .001) of surgery were significantly associated with ID.

**Conclusion:**

LESI increases the risk of ID in patients who undergo a subsequent lumbar discectomy within 6 months of injection.

## Introduction

The use of lumbar epidural steroid injection (LESI) in the treatment of chronic or subacute lower back pain and lumbosacral radiculopathy secondary to disc herniation and stenosis has been shown to be effective, despite a large degree of variation in reported efficacy.^1^ Whether through a transforaminal, caudal, or interlaminar approach, LESI remains one of the most commonly performed procedures in the United States.^2–4^ Though a relatively safe procedure, the rate of minor and major complications of LESI ranges from 0.07% to 9.6% in recent studies.^5–9^ These complications range from minor adverse effects such as flushing^10^ to rare major complications such as transient blindness.^11^ Other notable reported complications include discitis,^12^ increased index leg and back pain,^13^ epidural hematoma,^14^ and dural puncture.^8^

Nonetheless, there is little previous research on the association between LESI and subsequent intraoperative complications during spine surgery. In a recent study by Koltsov et al,^15^ about 25% of patients undergoing LESI for disc herniation or stenosis will go on to have a subsequent spine surgery. This significant percentage of patients warrants a closer look at the effects of steroid exposure on local spine tissues. One such possible long-term complication that has not been previously investigated is incidental durotomy (ID). ID is an undesirable intraoperative complication of spine surgery with a reported incidence of 1% to 17.4% in lumbar spine surgery.^16–19^The goal of this study was to investigate the association between LESI and ID in patients with a diagnosis of disc herniation undergoing a primary discectomy.

## Methods

A retrospective database review was conducted, using the commercially available PearlDiver Patient Records Database (www.pearldiverinc.com; PearlDiver Inc, Colorado Springs, CO), which contains all Medicare patient records from 2008 to 2014, searchable by International Classification of Diseases, 9th Revision, Clinical Modification (ICD-9-CM) diagnosis and procedure codes. As all data was de-identified, institutional review board approval was waived for this study.

First, all Medicare patients who underwent a primary lumbar discectomy (ICD-9 CM: 80.50, 80.51, 80.59) for a diagnosis of lumbar disc herniation and degeneration (ICD-9 CM: 722.10 722.52 722.93) from 2008 to 2014 were identified. Exclusion criteria included prior history of infection, trauma, primary malignancy or metastasis of the spine and same day arthrodesis. Our resulting cohort of 64 849 patients was then divided into 2 groups: patients who incurred an incidental dural tear and a proportionally matched (based on age, gender, and history of diabetes) control cohort of patients who did not have an ID.

From the same main cohort, we queried for all patients who had prior LESI (Current Procedural Terminology [CPT]: 64 484, 64 483, and 62 311) via both the interlaminar and transforaminal approach. This new cohort was further stratified into 4 mutually exclusive subgroups by duration between LESI and primary discectomy (<3 months, 3-6 months, 6 months to 1 year, and overall) and a comparison of the relative incidence of ID diagnosis was made amongst these subgroups. Patient characteristics including age, gender, Charlson Comorbidity Index (CCI), and pertinent prior comorbid diagnoses were queried and compared.

### Statistical Analysis

Patient characteristics were compared between cohorts using Welch’s *t* test and Pearson’s chi-square analysis. Pearson’s chi-square analysis was used to test for significant differences in categorical variables, including age groups, gender, comorbid medical conditions, and prior LESI.

Logistic regression analysis was used to analyze the association between LESI and ID. Initially, univariate analysis was performed on each assessed covariate. Then, a multivariate logistic regression analysis was employed to determine the independent relationship between LESI and ID using variables with *P* < .1 on univariate analysis. All results were considered significant at *P* < .05.

## Results

### Patient Demographics and Comorbidities

A total of 64 849 patients underwent a primary lumbar discectomy for a diagnosis of lumbar disc herniation and degeneration. Of these patients, 2369 incurred an ID while 62 480 patients did not experience an intraoperative ID. A summary of patient demographics and comorbidities is outlined in Table 1. There was no significant difference in CCI (5.72 vs 5.80, *P* = .159) between both groups. Additionally, patients in our dural tear group had a higher incidence of prior LESI.

**Table 1.** Patient Demographics.

Characteristic	No Dural Tear (N = 62 480)		Dural Tear (N = 2369; 3.7%)		*P^a^*
	n	%	n	%	
Age, years					
65-69	19 751	31.6	749	31.6	1.000
70-74	19 947	31.9	756	31.9	
75-79	14 392	23.0	546	23.0	
80-84	8390	13.4	318	13.4	
Female sex	31 525	50.5	1195	50.4	1.000
CCI	5.72 ± 2.34		5.80 ± 2.41		.159
Comorbidities					
Diabetes	26 444	42.3	1002	42.3	1.000
Obesity	10 573	16.9	397	16.8	.856
Tobacco history	18 674	30.0	730	30.8	.345
COPD	18 735	29.9	666	28.1	.054
Coagulopathy	4244	6.8	156	6.6	.724
CHF	8567	13.7	295	12.5	.085
Chronic kidney disease	7099	11.4	275	11.6	.736
Liver disease	5856	9.4	210	8.9	.425
Malnutrition	1363	2.2	50	2.1	.873
Rheumatoid arthritis	6109	9.8	247	10.4	.314
Prior history of ESI	16 958	27.1	830	35.0	**<.001**

Abbreviations: CCI, Charleston Comorbidity Index; COPD, chronic obstructive pulmonary disease; CHF, congestive heart failure; ESI, epidural steroid injection. ^a^Bold *P* values indicate statistical significance (*P* < .05).

### Association of Epidural Steroid Injection With Incidental Dural Tear

Overall incidence of ID was 3.7%. A greater proportion of patients within the ID group had a history of LESI (27.1% vs 35.0%, *P* < .001). Among the identified patients with a history of LESI, our analysis showed the incidence of ID was 4.9%, 5.0%, 3.8%, and 4.3% in our <3 month, 3-6 months, 6 months to 1 year, and overall prior LESI subgroups respectively (Table 2). When adjusted for selected covariates, prior LESI between 3 and 6 months (odds ratio [OR] 1.47, 95% confidence interval [CI] 1.20-1.81, *P* < .001) and within less than 3 months (OR 1.46, 95% CI 1.24-1.72, *P* < .001) prior to surgery were significantly associated with ID. Preoperative LESI outside of 6 months to the date of surgery had no significant association with ID. A multivariate analysis showed no significant association between the comorbid diagnosis chronic obstructive pulmonary disease (OR 0.92, 95% CI 0.84-1.00, *P* = .062), congestive heart failure (OR 0.93, 95% CI 0.84-1.03, *P* = .177), or liver disease (OR 0.89, 95% CI 0.79-1.01, *P* = .073) and ID (Table 3).

**Table 2.** Incidence of Dural Tear With Prior History of Epidural Steroid Injection.

Duration Prior to Surgery	Epidural Steroid Injection	Dural Tear	Incidence (%)
<3 months	8168	398	4.9
3-6 months	3554	176	5.0
6 months to 1 year	5280	202	3.8
Overall	19 435	830	4.3

**Table 3.** Dural Tear Association With Lumbar Epidural Steroid Injection and Other Risk Factors.^a^

Variable	Unadjusted		Adjusted	
	OR (95% CI)	*P*	OR (95% CI)	*P*
Age, years				
65-69	1.00 (0.92-1.09)	.996	—	—
70-74	1.00 (0.91-1.09)	.989	—	—
75-79	1.00 (0.91-1.10)	.988	—	—
80-84	1.00 (0.88-1.13)	.995	—	—
LESI				
<3 months	1.57 (1.41-1.76)	**<.001**	1.46 (1.24-1.72)	**<.001**
within 3-6 months	1.55 (1.33-1.81)	**<.001**	1.47 (1.20-1.81)	**<.001**
within 6 months to 1 year	1.16 (1.00-1.35)	**.042**	0.94 (0.79-1.11)	.443
Overall	1.45 (1.33-1.58)	**<.001**	1.15 (0.99-1.34)	.063
Comorbidity				
COPD	0.89 (0.82-0.97)	**.009**	0.92 (0.84-1.00)	.062
Congestive heart failure	0.90 (0.82-0.99)	**.038**	0.93 (0.84-1.03)	.177
Liver disease	0.88 (0.78-0.99)	**.035**	0.89 (0.79-1.01)	.073
Obesity	0.99 (0.91-1.09)	.905	—	—
Diabetes	1.00 (0.92-1.09)	.979	—	—
History of tobacco use	0.95 (0.87-1.03)	.204	—	—
Coagulopathy	0.95 (0.84-1.07)	.373	—	—
Chronic kidney disease	0.99 (0.90-1.09)	.840	—	—
Malnutrition	1.01 (0.87-1.17)	.921	—	—
Rheumatoid arthritis	0.95 (0.85-1.07)	.450	—	—

Abbreviations: CI, confidence interval; COPD, chronic obstructive pulmonary disorder; LESI, lumbar epidural steroid injection; OR, odds ratio. ^a^Bold *P* values indicate statistical significance (*P* < .05).

## Discussion

To our knowledge, this study is the first of its kind demonstrating the association of preoperative LESI with ID. A number of articles and reviews have analyzed the immediate and delayed effects of LESI on spine surgery, yet most have failed to highlight any association between LESI and ID.^5,7–9,20,21^ In the current study, we found that not only does a prior history of LESI increase the risk of ID in subsequent lumbar spine surgery, but this association occurs in a time-sensitive manner. Our results showed that patients who underwent a LESI within less than 6 months from day of surgery had increased odds of experiencing an intraoperative ID. It should be noted that outside of the 6-month preoperative window, LESI was not an independent risk factor for ID.

The adverse effects of corticosteroids on local tissue are well documented. In a basic science study on human connective tissue stem cells (hTSCs) treated with dexamethasone, Zhang et al^22^ demonstrated that hTSCs treated with high concentrations of dexamethasone had a decrease in cell proliferation and differentiation leading to a suppression of collagen expression and the formation of defective fatty and cartilage-like tissue that was more susceptible to rupture after implantation. Similarly, a 1967 study by Berlinder et al^23^ demonstrated that in vitro inoculation of fibroblast cells with a high concentration of corticosteroids resulted in diminished growth and eventually death of fibroblast cells. The most abundant extracellular matrix structural proteins of the dura are collagen types I, III, and IV synthesized by meningeal fibroblasts.^24^ The above stated inhibitory effect of corticosteroids on fibroblast growth and collagen production could help shed light on the possible etiology of our identified increased risk of ID with prior LESI. Future basic science research would be necessary to investigate this hypothesis.

A number of studies have investigated complications of LESI other than ID. Mandel et al^25^ showed that patients who had a prior history of LESI were 1.21 times more likely to incur a vertebral body fracture. Yang et al^26^ also demonstrated that prior LESI between 1 and 3 months (OR 1.8, 95% CI 2.3-4.6, *P* < .0001) and within less than a month (OR 3.2, 95% CI 2.3-4.6, *P* < .0001) of injection was significantly associated with 90-day postoperative infection following a lumbar decompression, further supporting the durational effects of steroid exposure reported in our study. Though these studies demonstrated an increased risk of complications associated with LESI, El-Yahchouchi et al,^8^ in a multicenter study of 16 638 consecutive patients, reported no associated major adverse events in patients with a history of LESI.

Preoperative risk factors for ID have been the focus of many studies.^27–30^ Buck et al^30^ reported an increased risk of ID in patients older than 73 years (OR 2.40, 95% CI 1.88-3.10, *P* < .0001). While our study did not show any significant difference in patient sex, Takahashi et al^28^ reported female sex to be another significant risk factor for ID in addition to degenerative spondylolisthesis, and juxtafacet cysts. A similar database analysis by Burks et al^31^ demonstrated that obesity was associated with increased incidence of ID. This result was not consistent with our findings as our analysis showed no significant association between obesity and ID. We reported no significant associated between diabetes and ID, contrary to findings in current literature.^32,33^ While our study only focused on first-instance primary discectomies to mitigate the confounding effects of revision surgery, it should be noted that revision surgery remains one of the most significant risk factors of ID. Patients undergoing revision surgery have been shown to have more than twice the odds (OR 2.21, 95% CI 2.63-2.98, *P* < .001) of ID.^27^

LESI still remains a relatively safe procedure. This is evident with the continued increase in utilization and widespread acceptance of LESI as a nonoperative management for lower back pain and lumbosacral radiculopathy.^34^ One of such benefits of LESI was demonstrated in a randomized controlled, double-blinded study by Riew et al.^35^ Results from this study demonstrated that the injection of anesthetic agent and betamethasone was associated with a significant proportion (59%) of previously identified surgical candidates avoiding surgery at a minimum of 5 years follow-up after LESI.^35^ Another similar study by Radcliff et al^36^ also reported an increase in rate of surgery avoidance in patient population treated with LESI compared with patients who did not receive LESI.

This study has a number of advantages. Our large sample size allowed for an appropriately powered analysis and effective comparison of patient characteristics, comorbidities, and outcome for clinically significant findings. In addition, we were able to identify and stratify any history prior of LESI into 3 mutually exclusive time periods to investigate the time-dependent effect of local steroid exposure on ID. Nonetheless, as any retrospective patient database review, our study faces its sets of limitations. The accuracy of our data is dependent on the accurate coding of the information when entered into patient medical records. Errors in coding, including instances of miscoding and noncoding of diagnoses, have been identified previously in national databases and are possible sources of error.^37^ In particular, preexisting comorbidities have been demonstrated to be underreported, and the ability of the present study to control for the confounding effects of comorbidities with regression analysis depends on the accurate reporting of those comorbidities.^38^ We were unable to study patient level clinical and surgical information such as needle size, level of injection, volume and concentration of steroid used, as well as the operating surgeon’s level of experience, which are all potential risk factors of ID.

## Conclusion

LESI increases risk of ID in patients who undergo a subsequent lumbar discectomy within 6 months of injection. Spine surgeons and pain specialists should be aware of this association for appropriate preoperative planning and scheduling. Extra precaution should be taken when operating on patients with a recent of history LESI.
